# 

**Published:** 1935

**Authors:** 


### CONTENTS.

| | Page |
|---|---|
| The Problem of Man's Origin. By A. Rendle Short, M.D., F.R.C.S. | 1 |
| Licence to Practise and Liberty to Teach Medicine in the English Provinces. By J. A. Nixon, C.M.G., M.D., F.R.C.P. | 19 |
| Habit and Illness. By H. H. Carleton, M.D., M.R.C.P. | 41 |
| Reviews of Books | 57 |
| Editorial Notes | 63 |
| Obituaries: Walter Salisbury, M.D., M.S., F.R.C.S. | 65 |
| Henry Waldo, M.D., C.M., M.R.C.P. | 66 |
| Meetings of Societies | 67 |
| The Medical Library of the University of Bristol | 69 |
| Publications Received | 72 |
| Local Medical Notes | 73 |

